# A Species Flock Driven by Predation? Secondary Metabolites Support Diversification of Slugs in Antarctica

**DOI:** 10.1371/journal.pone.0080277

**Published:** 2013-11-26

**Authors:** Nerida G. Wilson, J. Alan Maschek, Bill J. Baker

**Affiliations:** 1 Australian Museum Research Institute, The Australian Museum, Sydney, New South Wales, Australia; 2 Scripps Institution of Oceanography, University of California San Diego, La Jolla, California, United States of America; 3 Department of Chemistry, University of South Florida, Tampa, Florida, United States of America; 4 Center for Drug Discovery and Innovation, University of South Florida, Tampa, Florida, United States of America; Sars International Centre for Marine Molecular Biology, Norway

## Abstract

Antarctica's rich marine animal biodiversity has been substantially influenced by a complex glacial history, but it is unclear why some taxa responded with diversification while others did not. Despite being considered a single endemic sea slug species in the Southern Ocean, mitochondrial DNA sequencing of *Doris kerguelenensis* (Bergh, 1884) revealed a multitude of highly divergent lineages. But because of the uniparental inheritance of mitochondria, it was unclear whether those lineages represented a radiation of cryptic species or simply stochastic sorting patterns of populations that rarely reach equilibrium. Here we demonstrate that the mitochondrial groups in *D. kerguelenensis* also correlate with nuclear DNA. Additionally, by extracting secondary metabolites from the same individuals we sequenced, we were also able to directly link the secondary metabolome to a mitochondrial lineage. These metabolites are not derived from the diet, but instead are synthesized de novo and implicated in an anti-predatory role. The strong linkage between these metabolites and the mitochondrial lineages strongly suggests that these lineages represent cryptic species in an adaptive radiation. Over millions of years, episodic glacial cycles reduced the distribution of a formerly widespread slug into a series of small vicariant refuges, vulnerable to genetic drift and predation pressure. The recognition of this marine invertebrate species flock implicates a strongly synergistic role for selection and allopatry driving speciation in this system.

## Introduction

There is no doubt that intense selection has influenced marine community structure in Antarctica [1]–[3]. The absence or rarity of many major animal groups (e.g. cartilaginous fish, brachyuran crabs) is linked to traits that favour survival in the physiologically-challenging conditions [4]. However, high levels of biological diversity in Antarctica have generally been attributed to repeated allopatric speciation during glacial cycles, known as a ‘biodiversity pump’ [2], [5]. Thought to be the most profound factor driving evolution in the Southern Ocean, the Antarctic Biodiversity Pump hypothesis proposes that Milankovitch-driven glacial cycles drove range expansion and contraction that increased overall speciation rates. During glacial cycles, grounded ice shelves have extended far from the continent, massively disrupting the Antarctic continental shelf and slope as habitable areas for marine benthic organisms [6]. Despite these widespread extinctions, some ice-free shelf refugia can be inferred [7]. These would have sustained reduced population sizes for previously widespread species, and simultaneously provided barriers between populations, facilitating speciation by allopatry.

A large group of putatively cryptic species (herein referred to as phylogroups) within the Antarctic morpho-species *Doris ‘kerguelenensis’* (Bergh, 1884) was recently proposed, based on mitochondrial DNA data [8]. These large sea slugs show reduced dispersal potential because they lack a free-swimming larval stage and can only crawl along the benthos as adults [9], [10]. Quantitative sampling has shown that this species is restricted to continental shelf and slope regions in the Southern Ocean [11]. With these traits, we would predict the ‘biodiversity pump’ to produce many species from a once widespread ancestral stock, or minimally, that population structure would be genetically-subdivided with high levels of geographic concordance [12]–[15]. Instead, many of these mitochondrially-elucidated phylogroups (PGs) are known to show broad distributions, e.g. >4000 km (PG24, PG29), and a large number occurred in sympatry [8]. If each phylogroup represented a cryptic species, it would imply higher-than-expected rates of dispersal.

In addition to the diverse phylogroups in *Doris ‘kerguelenensis’*, natural products surveys have also recovered a wide variety of secondary metabolites from this species [16]–[18]. These are of medical interest since several tested compounds can inhibit a form of human leukemia [18]. In dorid nudibranchs, these chemicals are synthesized *de novo*, and appear to be independent of diet [19], [20]. These chemicals could represent phenotypic differences among the phylogroups, and if confirmed, would provide additional support that the phylogroups in *D. ‘kerguelenensis’* actually represent different species.

To fulfill the criteria of a core species flock [21] that satisfies evolutionary criteria, the mitochondrial lineages in *D. ‘kerguelenensis’* would have to be confirmed as putative species, and to represent a single, species-rich, endemic evolutionary radiation. Recognition as a full species flock also requires a demonstration of ecological diversity and habitat dominance [21]. This addition of ecological relevance makes a full species flock almost synonymous with an adaptive radiation since ecological criteria feature strongly in adaptive radiation criteria. Although the speed at which the diversification has occurred is arguably unimportant [22], a relationship between phenotypic traits and environment, and fitness benefits from those traits are key to testing adaptive radiations [22], [23]. Here we set out to test whether nuclear data was concordant with the mitochondrial lineages of previous work, which would support the concept of a cryptic species radiation. We then assessed whether there was any secondary metabolite differences among these putative species, which may confer evolutionary advantages.

## Materials and Methods

### Sample collection

Animals for genetic and chemical analyses were collected by SCUBA from the surrounds of Palmer Station, Antarctic Peninsula (2008/2009) (n = 54) (PSC08-06). Each animal was photographed, weighed and had a tissue subsample fixed in 95% ethanol for molecular analyses, before being frozen at −80°C. 115 additional specimens from a previous study [8] were newly sequenced for an additional nuclear gene (Adenine Nucleotide Transporter, ANT), but were unexamined for metabolite profiles. Those specimens were collected predominantly from the Scotia Sea and Antarctic Peninsula area, but also included a few representatives from South America, the Ross Sea and the Weddell Sea [8]. GenBank accession numbers are JX680531-JX680589, JX683455-JX683512, KC246596-KC246770, and all sample metadata are available in electronic supplementary material Table S1.

### Ethics statement

Collection occurred in an area south of 60°S latitude, covered by the Antarctic Treaty, which establishes freedom of scientific investigation. No specially-protected areas or organisms were utilised, and no additional permits were required. The region does not include private property.

### Liquid Chromatography/Mass Spectrometry (LC/MS) sample preparation

Frozen nudibranchs were thawed and extracted with CH_2_Cl_2_:MeOH (1∶1; 3X for 24 h) to generate an external (whole organism) extract, which was concentrated under reduced pressure. The solid was reconstituted in EtOAc and partitioned against H_2_O (3X); the organic layer was removed and concentrated under reduced pressure. Each extract was filtered over a small plug of silica gel and eluted with EtOAc. The extract was again reduced and resuspended in MeOH and filtered over SEP-PAK C18-RC cartridges (Waters). After reduction, each extract was resuspended in CHCl_3_ then diluted in MeOH for LC/MS analysis.

### Chromatography and mass spectrometric analysis

Chromatography was performed using an Agilent 1100 Series LC/MS equipped with an Agilent LC/MSD VL electrospray ionization mass spectrometer. Injections of nudibranch extract (20 µL; 0.25 mg/mL in MeOH) were made onto an Agilent Eclipse XDB-C18 column (150×4.6 mm, 5 µm). The column was maintained at 25°C and eluted under gradient conditions at a flow rate of 1.0 mL/min; mobile phase component A consisted of 100% H_2_O (0.05% TFA), and B of 100% acetonitrile (0.05% TFA). The column was linearly increased from 40% B to 100% B over 11 min and maintained at 100% B for 4 min. The mass spectrometer was operated in positive ion mode, with a capillary voltage of 3 kV, the nebulizer gas pressure was set to 35 psig, fragmentor set to 70 V, spray chamber gas temperature of 350°C, and a drying gas flow of 10 L/min. The resolving power was set to provide unit-mass resolution across the entire range m/z 200–500. Data were collected from 0–17 min.

### Chemometric analysis

The LC/MS chromatograms were converted to netCDF file format and transferred to MSMetrix (Maarssen, The Netherlands, http://www.msmetrix.com/) for clustering analyses using their proprietary software package MSXelerator. In the module MSCompare, all multivariate analysis techniques (dendrogram, principle component analysis (PCA)) utilized extracted baseline corrected total chromatogram mass (TCM) spectra where each *m/z* value of interest (*m/z* 200–500) is summed at that nominal value. Doing this for all *m/z* values with a RT of 2–17 min creates a mass spectrum with, in this case, 300 *m/z* values along the x-axis. MSMetrix software module MSCompare generates clustering coefficients based on comparison of TCM spectra shapes to classify animals based on TCM similarities.

### Molecular data

Total genomic DNA was extracted from ethanol-fixed samples using a DNeasy Blood & Tissue Kit (Qiagen), according to manufacturer's instructions. 1–4 ul of genomic extract was added to illustra PuRe Taq RTG polymerase chain reaction (PCR) beads (GE Healthcare) to amplify the selected genes. Partial Cytochrome Oxidase I (COI) was amplified using published primers [24] and a standard ‘barcoding’ protocol that utilizes 5 cycles of 45°C followed by 40 cycles of 51°C. Partial 16S ribosomal RNA was amplified using primers [25] annealing at 50°C for 35 cycles. Partial sequences of adenine nucleotide (ADP/ATP) translocase (ANT) were amplified using the F and R1 combination [26] annealing at 39°C for 5 cycles, followed by 40 cycles of 48°C. Subsequently, specific primers were developed AusANTF (5′- CAACACAGGCCCTCAATTTT-3′) and AusANTR (5′-TTTCATCAAAGGACATGAAGC-3′), with the first 5 cycles annealing at 55°C, and the subsequent 35 cycles annealing at 50°C. PCR products were purified directly using ExoSAP-IT (GE Healthcare) or by gel purification using Montage DNA Gel Extraction Kits (Millepore). Products were sequenced for bi-directional reads, analysed with an ABI PRISM 3730 (Applied Biosystems, Inc.) sequencer. Sequences were concatenated and edited using Sequencher 4.8 (Gene Codes Corporation). COI and ANT data were unambiguously aligned by eye in Se-Al (no insertions required), and were checked for stop codons by translation. 16S was aligned with MAAFT [27] (Multiple Alignment using Fast Fourier Transform) using the Q-INSI-I option to best accommodate secondary structure (v 6.0; online http://align.bmr.kyushuu.ac.jp/mafft/software/). Three of the 175 ANT sequences showed a single nucleotide polymorphism (SNP) at one of two sites, which were coded as ambiguous for phylogenetic analyses.

### Phylogenetic analyses and indices

Evolutionary models were inferred for each gene partition using jModelTest2 [28] implementing the Akaike Information Criterion (ANT: HKY+I; 16S: TPM2uf+I+G; COI: TIM2+I+G;). The final combined data set consisted of 1603 characters (ANT: 515 bp; 16S: 461 bp; COI: 627 bp). Any gaps in the combined data set were treated as missing data, and no part of the 16S alignment was masked or excluded (only one shared indel was present in the ingroup).

First, unrooted maximum likelihood analyses on the ANT data set were carried out in RAxML [29] v7.2.8 implemented in the raxmlGUI [30] v1.2 to compare to groups identified by COI data. We used a GTR+G model as suggested by Stamatakis in the RAxML manual 2.2.3 to avoid the correlation of the proportion of invariant sites with the alpha parameter of the gamma distribution, and we assessed clade support with 1000 ‘thorough’ bootstrap replicates.

Secondly, to be included in the combined data phylogenetic analyses, individuals had to be represented by data for two of the three genes. Six outgroups were selected in total. Two species of *Doris* from the northern hemisphere and two additional generic exemplars were selected from the morphologically-defined Dorididae *sensu* [31]. Additionally, two representatives from the Cadlinidae *sensu* [32] which are sister to Dorididae+Discodorididae [31], provided rooting for the trees. Outgroups were represented by existing and new data for 16S and COI respectively- *Doris pseudoargus* (AF249224, AY345030), *Doris montereyensis* (KC153024, KC153022), *Conualevia alba* (KC153023, KC153021), *Doriopsis granulosa* (AF249223, AF249798), *Aldisa banyulensis* (AY345039, AY345039) and *Cadlina laevis* (EU982766, EU982716). No ANT data was available for outgroups.

The three-gene dataset was analysed in RAxML as described above, with data partitioned by gene, using joint branch parameter estimation, and 1000 bootstrap replicates. The same dataset was analysed in MrBayes v3.1.2 [33] with default priors and unlinked partitions for parameter estimation. Eight iterations of 20 million generations were run with 6 chains sampling every 1000 generations, and a consensus built from trees remaining after 10% burn-in was removed (n = 144,008).

## Results and Discussion

### Concordance of mitochondrial and nuclear lineages supports species diversification

To investigate if phylogroups represented stochastic mitochondrial patterns rather than cryptic species, we sequenced a nuclear protein coding gene Adenine Nucleotide Transporter (ANT) for specimens in known mitochondrial phylogroups [8]. An unrooted maximum-likelihood tree of ANT data shows that each cluster corresponds to one or more mitochondrial phylogroups, and that there are no conflicting nuclear-mitochondrial signals from the data (electronic supplementary material, figure S1). Most nuclear ANT clusters corresponded to a single mitochondrial phylogroup (65%), some to two combined phylogroups (17%), and some to 4–6 combined phylogroups (18%). In these instances the nuclear data could not differentiate among mitochondrial groups but that is expected since nuclear genes have a lower mutation rate [34]. The concordance demonstrated here suggests the mitochondrial groupings in this sea slug are not stochastic, but instead reflect speciation patterns.

### Concordance of genetic and chemical diversity supports ecological diversification

**Figure 1:**
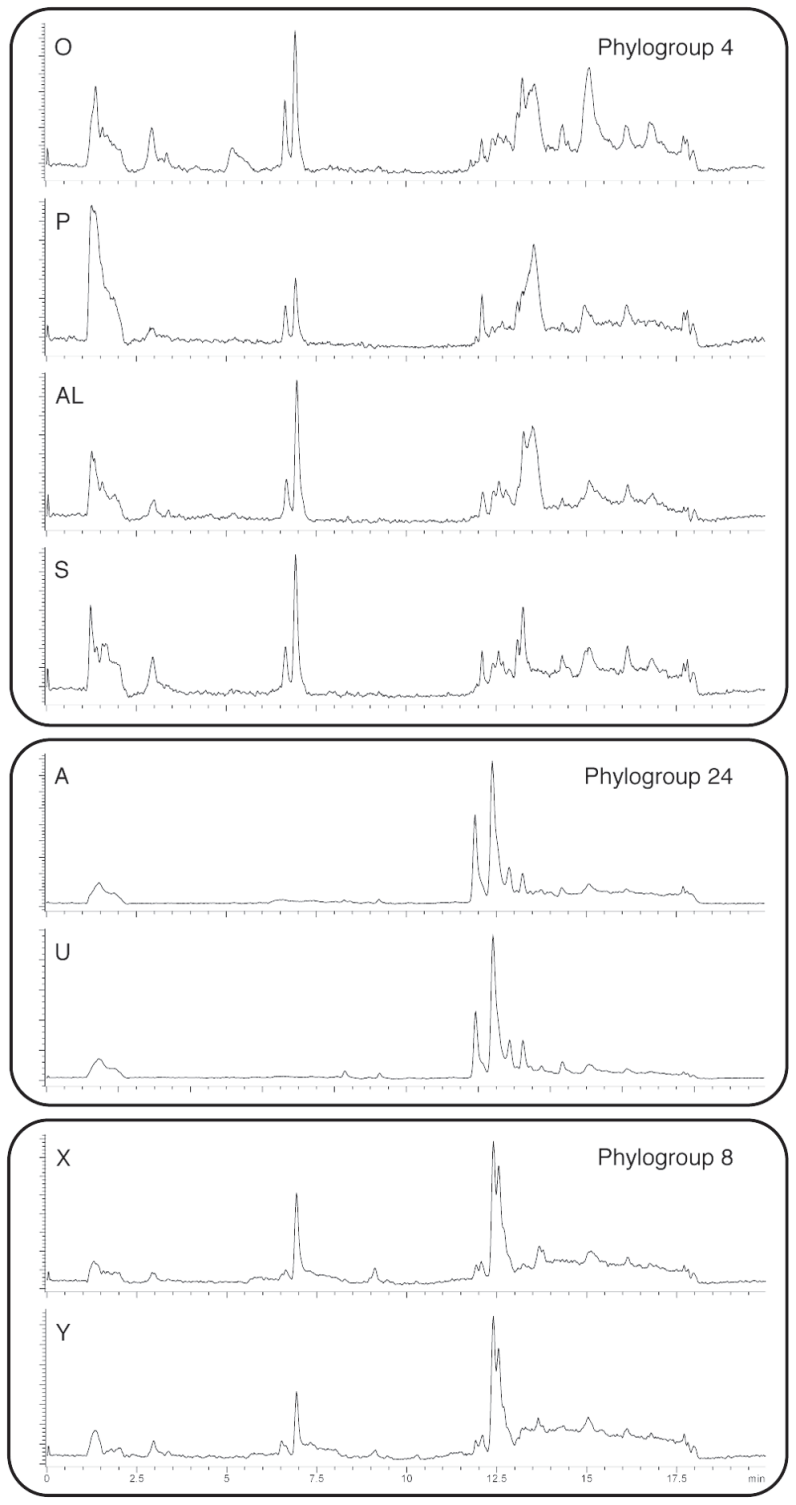
Examples of total ion current (TIC) chromatogram mass spectra for selected phylogroups. Individual slugs are identified with alphabetic characters; *m/z* values show a retention time of 2.5–17 min.

**Figure 2:**
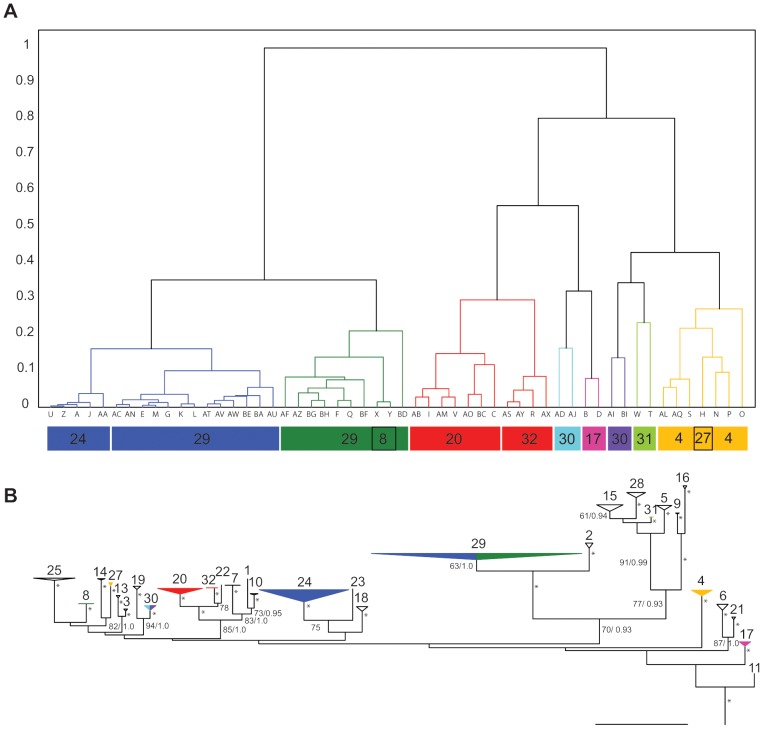
Group membership based on chemical and genetic data. (**a**) Multivariate dendrogram (similarity metric  =  correlation coefficient) for 54 individuals of *Doris ‘kerguelenensis’* based on extracted total chromatogram mass (TCM) spectra for *m/z* values with a retention time of 2.5–17 min. The correlation is not size dependent, but rather takes the baseline corrected average mass spectrum of a sample so that comparisons of shapes are more important. Groups with similar chemistry are designated and coloured based on an arbitrarily designated cut-off threshold of 0.3 similarity. Letters below the dendrogram refer to individual slugs. Numbers in coloured blocks below dendrogram give the corresponding phylogroups derived from molecular data. (**b**) Maximum-likelihood (ML) topology for 193 individuals of *D. ‘kerguelenensis’* based on combined mitochondrial COI, 16S and nuclear ANT data. *indicates bootstrap support of 95 or above *and* posterior probability of 0.98 or above. Support values less than 60 or 0.90 are not shown. Terminals are coloured to show the distribution and designation of individuals with corresponding chemical data. Scale bar, 0.07 expected changes per site, ML.

A literature survey of compounds recovered from *D. ‘kerguelenensis’* described four types of diterpenoid carbon skeletons consisting of 33 compounds (electronic supplementary material, Table S2). We characterised the secondary metabolome from 54 individual slugs from the Palmer Archipelago, Antarctic Peninsula using liquid chromatography mass spectroscopy (LC/MS). Slugs were easily grouped according to the appearance of their total ion chromatograms (Fig. 1). When this data was converted into quantifiable multivariate statistics, a dendrogram of correlation coefficients showed clearly chemically-defined clusters (Fig. 2A).

To test the hypothesis that the genetic and chemical diversity present in *D. ‘kerguelenensis’* were linked, we sequenced the same 54 slugs for the barcoding region of Cytochrome Oxidase I (COI), partial 16S ribosomal DNA and the nuclear ANT gene. We combined our novel sequence data with available 16S and COI data (and additionally generated ANT data for these individuals) and generated a new phylogenetic hypothesis for the group (n = 193 individuals) (Fig. 2B, electronic supplementary material, figures S2, S3). All *D. kerguelenensis* formed a monophyletic group. We also found three previously unknown lineages (designated phylogroups 30–32), which supports the prediction that there are still undiscovered lineages in well-sampled areas [8]. Unsampled areas, such as East Antarctica, are likely to yield even more lineages. All phylogroups (with the exception of PG15, PG29) were highly statistically-supported in all analyses. Many of the basal nodes are unsupported (essentially forming a polytomy), which is not unexpected if their radiation was linked to a series of glacial vicariant events over a short time scale. Congruent with the results from the nuclear ANT data, we found that there was a notably high level of correlation between mitochondrially-identified groups and those that shared a suite of chemical compounds (Fig. 2).

**Figure 3:**
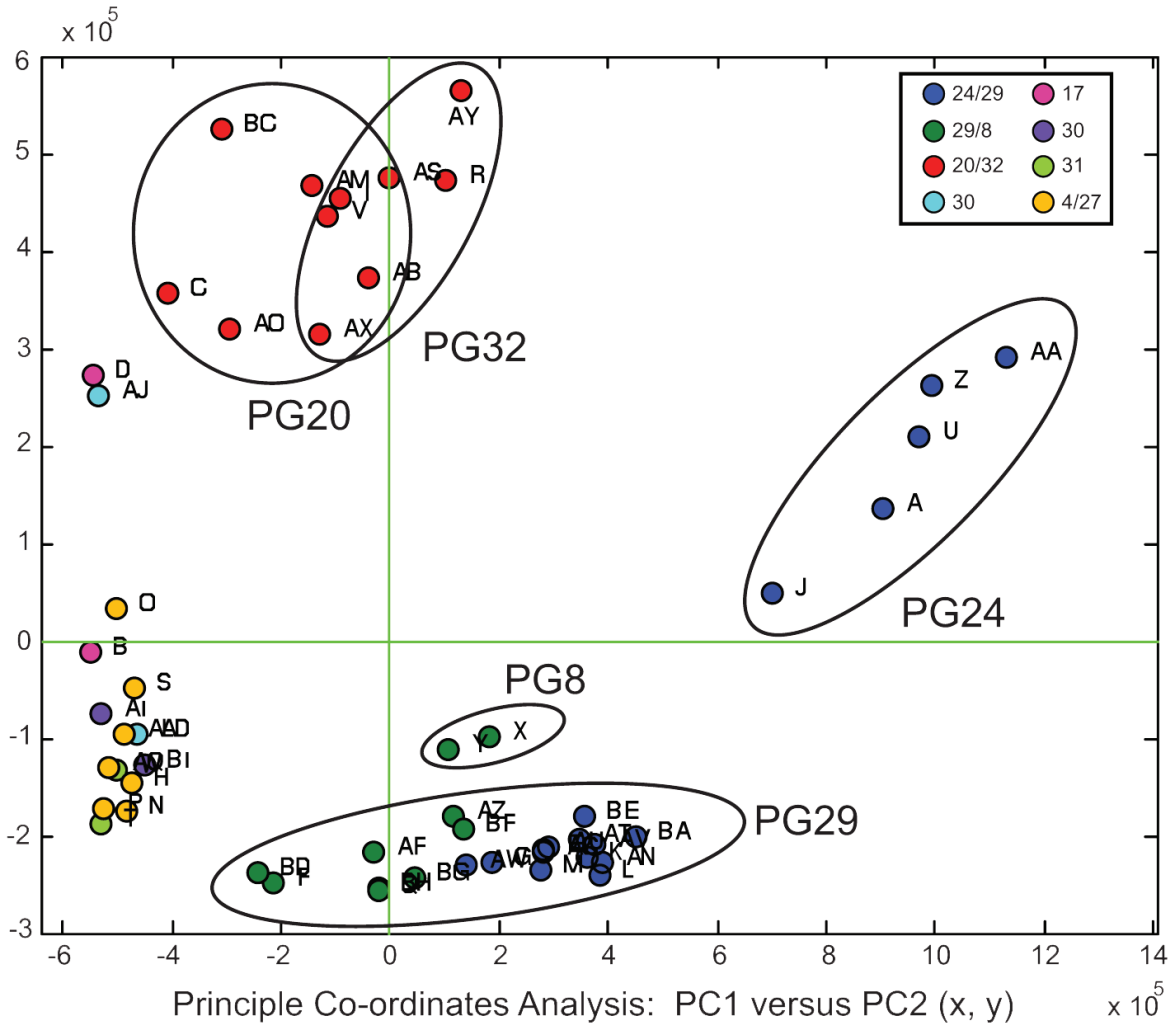
Principal component analysis of TCM spectra, PC1versus PC2 (x,y; centering applied) for selected phylogroups. Data based on extracted total chromatogram mass (TCM) spectra for *m/z* values with a retention time of 2.5–17 min. Each dot represents an individual slug coloured according to its chemo-group designation from the dendrogram in Figure 2. The genetic phylogroup designations are overlaid for select groups, and demonstrate that some putative chemical differences within a phylogroup identified by similarity analyses are not corroborated by PCA (e.g. phylogroup 29). It also shows that different phylogroups with similar chemical profiles can de differentiated by PCA (e.g. phylogroups 24 and 29).

Putative exceptions to the genetic-chemical correspondence in Fig. 2 appeared to occur in two ways. Some phylogroups contained individuals that exhibited different chemical signatures (PG30, PG29). Additionally, some distantly-related phylogroups showed the same chemical arsenal (e.g. PG24+PG29; PG29+8). Examining variation in the LC/MS data in three-dimensional space via principal components analysis (PCA) clarified these potential exceptions (Fig. 3). The two chemo-groups encompassed within phylogroup 30, and the two within phylogroup 29, overlap in PCA and cannot be easily differentiated (although the PG30 sample size is small and is not encircled). Similarly, the similar chemical suites found in PG24+29 and PG29+8 can easily be differentiated in PCA, suggesting these are cases of convergent metabolite acquisition. Conversely, in the only instance where we have both chemical and genetic data for closely-related lineages (PG20+32, Fig. 2b) we see that these groups cannot be differentiated with PCA (Fig. 3). This supports the concept of a suite of secondary metabolites with a single origin.

### A species flock generated by vicariance and selection

A scenario that could account for: 1) a high correlation between genetic and chemical groups; 2) evidence for convergent acquisition of a suite of metabolites and 3) evidence for a single origin for other metabolite signatures in *D. ‘kerguelenensis’*, would likely include strong selection pressure during times of retreat into glacial refugia. There is already experimental evidence that the secondary metabolites expressed by the slugs elicit anti-predator responses to generalist predators like sea stars [35]. The diversity of natural products found in *D. ‘kerguelenensis’* corroborates the screening/molecular promiscuity hypothesis [36]–[38], which predicts that an organism maximizes its survival probability by producing high levels of chemical diversity.

Because an organism's metabolome is a direct response to its environment, examining the particular role that metabolites play can help elucidate the system-wide influences that shape that metabolome [39]. Plausibly, different shelf refugia would have contained different predators, so we would predict strong selection for a predator-specific metabolomic signature. Depending on the length of isolation within a refuge, it is reasonable to assume that slugs without the right defensive measures would become extinct. This type of selection pressure, coupled with genetic drift for small populations, should result in rapid fixation of haplotypes concordant with the evolutionary patterns revealed here. In times of glacial retreat, refugia would open, and individual slugs would be free to disperse more widely. Because the predators would do the same, a diversity of slugs and their compounds should be maintained in the environment until the next glacial period. Then, inside each refugium the predation ( = selection) experiment would be repeated again. During interglacials and periods of secondary contact, mechanisms such as co-adaptation of mitochondrial and nuclear genomes [40] might also act to disadvantage hybrids. It is also likely that chemical recognition cues for mate choice work in the same way; other dorids have been shown to discriminate between conspecific and heterospecific individuals using chemical cues left in mucous trails [41].

Without invoking a dual role for selection and genetic drift, it is hard to account for the strong correlation of the secondary metabolome with both the mitochondrial and nuclear genome. The scenario proposed here for *D*. ‘*kerguelenensis’* can also account for different evolutionary lineages acquiring the same set of secondary metabolites (different lineages being exposed to the same predators in independent refugia) and a homologous origin of other metabolomic suites (slugs with shared ancestry subsequently split into different refugia with the same predators). Developing evolutionary hypotheses for organisms with bioactive metabolites is important, since chemical diversity and activity can be highly correlated with phylogeny [42], which is useful to create a predictive framework for bioactivity.

Our results suggest that repeated glacial cycling produced a large evolutionary radiation in what was previously recognized as a single species of sea slug [43]. The lack of geographical correlation within the Southern Ocean for these cryptic species suggests significant post-glacial dispersal for an organism that lacks a larval phase. *Doris ‘kerguelenensis’* certainly satisfies the evolutionary criteria of a core species flock [21] in that it is endemic to the Southern Ocean, monophyletic and species rich. It is clear that marine species flocks are no longer as rare as once thought [21]. By demonstrating the differences in secondary metabolite profiles in the *D. ‘kerguelenensis’* species complex, we also fulfill the criteria of ecological diversity.

Traditionally, adaptive radiations have involved efficient exploitation of ecological opportunity [23], usually during expansion into new and unoccupied habitats e.g. [44], whereas in our study it appears that the radiation is strongly driven by predation. Previous debate about the role of predation in adaptive radiations often centered on whether predation affected resource competition by reducing prey density [45], particularly in the classic cases of crypsis and mimicry e.g. [46]. Some experimental support was found for predation as a direct source of selection in terrestrial systems where interspecific competition for resources is weak [47]. Our results increase that generality to marine, non-visual predator-prey systems in a hybrid of the “spontaneous clusterization” and “invasion of empty niches' dynamic models of adaptive radiation [48].

Although there is no question that secondary metabolites are a strong defense against predation and can confer fitness benefits, whether this system truly represents an adaptive radiation requires further experimental manipulation. It is difficult to argue that being forced into a glacial refugium truly represents an ecological ‘opportunity’. The only difference from the usual habitat is a reduction in range, and smaller populations sizes with accompanying stochastic community effects. However, nonecological speciation is still possible in an adaptive radiation if different advantageous alleles are fixed in isolated populations in similar environments [23].

Regardless, the phylogroups previously recognized in *Doris ‘kerguelenensis’* clearly represent a cryptic species complex, with unknown species still being discovered. If niche diversification [49] and non-planktonic development [50] are thought to form a ‘one-two punch’ promoting speciation [14], we also demonstrate that in this system, selection may have provided the final blow to shatter one previously widespread species into a newly-recognized species flock in the Southern Ocean.

## Supporting Information

Figure S1 **Unrooted maximum-likelihood tree based on adenine nucleotide transporter (ANT) data.** Phylogroup numbers from mitochondrial data overlaid onto ANT clusters. Node support based on 1000 bootstrap replicates.
(TIF)

Figure S2 **Maximum-likelihood tree based on combined COI, 16S and ANT dataset.** Phylogroup numbers from mitochondrial data are overlaid. Node support based on 1000 bootstrap replicates.
(TIF)

Figure S3 **Bayesian inference tree based on combined COI, 16S and ANT dataset.** Based on 8 iterations of 20 million generations (6 chains sampling every 1000 generations), 10% burn-in removed.
(TIF)

Table S1 **Sample metadata for individual** ***Doris kerguelenensis*** **included in this study.**
(PDF)

Table S2 **Geographical and depth distribution of diterpenes found in** ***Doris kerguelenensis*** **.** Synonymous compounds are reported in rounded parentheses, and geographical detail in square parentheses.
(PDF)
