# Supplementary figures and images for: A Species Flock Driven by Predation? Secondary Metabolites Support Diversification of Slugs in Antarctica
Source: PLoS One. 2013 Nov 26;8(11):e80277. doi: 10.1371/journal.pone.0080277 (PMC3841181; doi:10.1371/journal.pone.0080277)

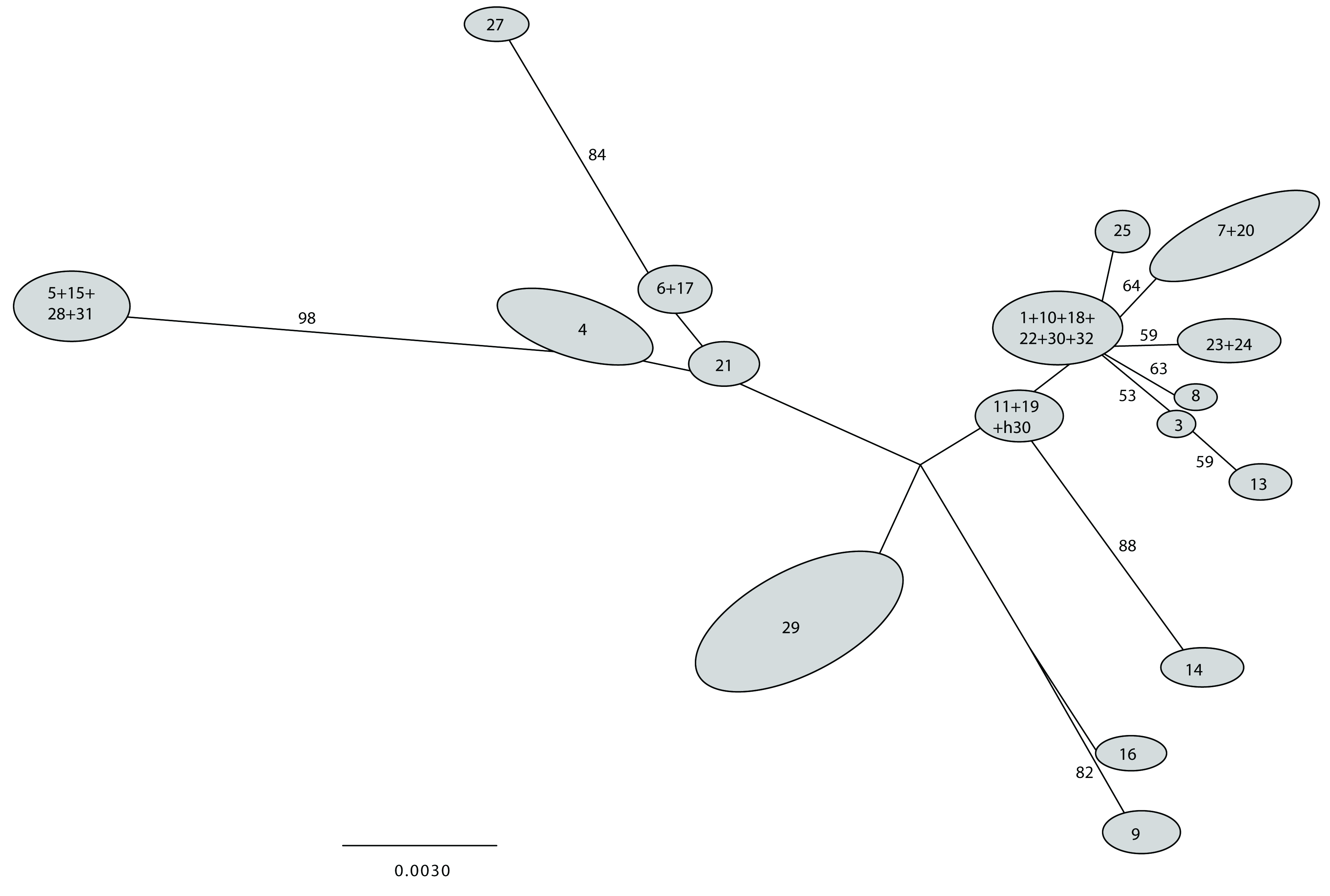

Supplement: Figure S1 — Unrooted maximum-likelihood tree based on adenine nucleotide transporter (ANT) data. Phylogroup numbers from mitochondrial data overlaid onto ANT clusters. Node support based on 1000 bootstrap replicates. (TIF) [file pone.0080277.s001.tif]
