# Supplementary material for: A Species Flock Driven by Predation? Secondary Metabolites Support Diversification of Slugs in Antarctica
Source: PLoS One. 2013 Nov 26;8(11):e80277. doi: 10.1371/journal.pone.0080277 (PMC3841181; doi:10.1371/journal.pone.0080277)
Supplement: Table S1 — Sample metadata for individual Doris kerguelenensis included in this study. (PDF) [file pone.0080277.s004.pdf]

Supplementary Table 1. Sample metadata for individual *Doris kerguelenensis* included in this study.

Lab code	Locality	Station	Lat	Long	Collection date	Collector	Cruise/collection	Museum voucher	Depth (m)	Phylogroup	Genbank COI	Genbank 16S	Genbank ANT
CAS IZ171180a	Burdwood Bank	9-BT7	S54°31'12''	W56°37'12''	22-May-04	Susie Lockhart	ICEFISH	CAS IZ171180a	125	1	EU823130	EU823221	KC246596
ZSM2002 1065-1	Burdwood Bank	PS61/145-1	S54°01.36'	W62°01.33'	5-Apr-02	Katrin Linse	LAMPOS	ZSM2002 1065-1	272	2	EU823131	EU823223	
ZSM2002 1065-2	Burdwood Bank	PS61/145-1	S54°01.36'	W62°01.33'	5-Apr-02	Katrin Linse	LAMPOS	ZSM2002 1065-2	272	2	EU823132	EU823223	
ZSM2002 1065-3	Burdwood Bank	PS61/145-1	S54°01.36'	W62°01.33'	5-Apr-02	Katrin Linse	LAMPOS	ZSM2002 1065-3	272	2	EU823133	EU823223	
ZSM2002 1048-1	Herdman Bank	PS61/217-1	S59°54.98'	W32°28.33'	18-Apr-02	Michael Schrödl	LAMPOS	ZSM2002 1048-1	520	3	EU823135	EU823226	KC246598
ZSM2002 1048-2	Herdman Bank	PS61/217-1	S59°54.98'	W32°28.33'	18-Apr-02	Michael Schrödl	LAMPOS	ZSM2002 1048-2	520	3	EU823136	EU823227	KC246597
CAS IZ171171	South Sandwich	52-BT38	S58°56'60''	W26°27'36''	18-Jun-04	Susie Lockhart	ICEFISH	CAS IZ171171	85	4	EU823137	EU823228	KC246599
PSC08-06-AL	western Peninsula	Jannus	S 64 47.107'	W 64 06.125'	8-Feb-08	Bill Baker & Alan Maschek	SCUBA	destroyed	0-35	4	JX680534	JX683458	KC246604
PSC08-06-N	western Peninsula	**Bonaparte**	S 64 46.662'	W 64 03.986'	13-Jan-08	Bill Baker, Craig Aumack & Alan Maschek	SCUBA	destroyed	0-35	4	JX680531	JX683455	KC246600
PSC08-06-O	western Peninsula	**Bonaparte**	S 64 46.662'	W 64 03.986'	13-Jan-08	Bill Baker, Craig Aumack & Alan Maschek	SCUBA	destroyed	0-35	4	JX680532	JX683456	KC246601
PSC08-06-P	western Peninsula	**Bonaparte**	S 64 46.662'	W 64 03.986'	13-Jan-08	Bill Baker, Craig Aumack & Alan Maschek	SCUBA	destroyed	0-35	4	JX680533	JX683457	KC246605
PSC08-06-S	western Peninsula	Gamage	S 64 46.476'	W 64 03.415'	18-Jan-08	Alan Maschek & Craig Aumack	SCUBA	destroyed	0-35	4	JX680536	JX683460	KC246602
PSC08-06-AQ	western Peninsula	Litchfield	S 64 45.972'	W 64 05.964'	10-Feb-08	Bill Baker & Alan Maschek	SCUBA	destroyed	0-35	4	JX680535	JX683459	KC246603
ZSM2002 0797	Elephant Is.	PS61/047-1	S61°04.18'	W54°36.81'	30-Jan-02	Katrin Linse	ANDEEP 1	ZSM2002 797	190	5	EU823139	EU823230	KC246607
G326.5	Bransfield	45-41	62°43.535S	55°11.224W	5-Mar-06	Susie Lockhart & Vincent Smith	AMLR 2006 - Leg II	USNM 1120709	167	5	EU823175	EU823230	KC246609
G317.1	Bransfield	35-19	62°28.906S	56°17.261W	25-Feb-06	Susie Lockhart & Vincent Smith	AMLR 2006 - Leg II	USNM 1121340	344	5	EU823145	EU823230	KC246606
G324.8	Bransfield	19-11	63°00.022S	58°05.010W	22-Feb-06	Susie Lockhart & Vincent Smith	AMLR 2006 - Leg II	USNM 1120714	235	5	EU823177	EU823230	KC246608
G324.9	Bransfield	19-11	63°00.022S	58°05.010W	22-Feb-06	Susie Lockhart & Vincent Smith	AMLR 2006 - Leg II	USNM 1121336	235	5	EU823175	EU823230	
G316.2	nr Elephant Is	74-32	61°48.831S	54°00.131W	2-Mar-06	Susie Lockhart & Vincent Smith	AMLR 2006 - Leg II	USNM 1121304	291	6	EU823143	EU823233	KC246611
G131.9	Bransfield	Station 49	S63°13'45''	W58°45'20''	6-Dec-04	Nerida Wilson	LMG04-14	USNM 1121595	87	6	EU823186	EU823254	KC246610
PSC08-06-AP	western Peninsula	Litchfield	S 64 45.972'	W 64 05.964'	10-Feb-08	Bill Baker & Alan Maschek	SCUBA	destroyed	0-35	6	JX680538	JX683462	KC246612
PSC08-06-BM	western Peninsula	Litchfield	S 64 45.972'	W 64 05.964'	5-Mar-08	Bill Baker & Charles Amsler	SCUBA	destroyed	0-35	6	JX680537	JX683461	KC246613
G326.6	Bransfield	45-41	62°43.535S	55°11.224W	5-Mar-06	Susie Lockhart & Vincent Smith	AMLR 2006 - Leg II	USNM 1120724	167	7	EU823167	EU823242	KC246618
G325.3	Bransfield	76-35	62°49.005S	56°39.477W	3-Mar-06	Susie Lockhart & Vincent Smith	AMLR 2006 - Leg II	USNM 1120711	108	7	EU823167	EU823242	KC246616
G325.6	Bransfield	76-35	62°49.005S	56°39.477W	3-Mar-06	Susie Lockhart & Vincent Smith	AMLR 2006 - Leg II	USNM 1120706	108	7	EU823167	EU823242	KC246617
G253.11	Bransfield	21-192	S 63°08.838'	W 57°07.441'	25-May-06	Nerida Wilson	LMG06-05	USNM 1121617	146	7	EU823167	EU823242	KC246615
G131.3.02	Bransfield	Station 49	S63°13'45''	W58°45'20''	6-Dec-04	Nerida Wilson	LMG04-14	USNM 1121618	87	7	EU823167	EU823242	KC246614
G319.2	Bransfield	39-52	62°30.875S	55°58.886W	9-Mar-06	Susie Lockhart & Vincent Smith	AMLR 2006 - Leg II	USNM 1121346	238	8	EU823151	EU823237	KC246619
G321.1	Bransfield	87-54	62°44.859S	57°33.042W	10-Mar-06	Susie Lockhart & Vincent Smith	AMLR 2006 - Leg II	USNM 1120703	430	8	EU823151	EU823237	KC246622
G131.2	Bransfield	Station 49	S63°13'45''	W58°45'20''	6-Dec-04	Nerida Wilson	LMG04-14	lost	87	8	EU823151	EU823237	KC246623
PSC08-06-X	western Peninsula	Hermit	S 64 48.139	W 64 1.438	29-Jan-08	Bill Baker & Alan Maschek	SCUBA	destroyed	0-35	8	JX680539	JX683463	KC246621
PSC08-06-Y	western Peninsula	Hermit	S 64 48.139	W 64 1.438	29-Jan-08	Bill Baker & Alan Maschek	SCUBA	destroyed	0-35	8	JX680540	JX683464	KC246620
G319.3	Bransfield	39-52	62°30.875S	55°58.886W	9-Mar-06	Susie Lockhart & Vincent Smith	AMLR 2006 - Leg II	USNM 1121332	238	9	EU823152	EU823238	KC246624
G324.5	Bransfield	19-11	63°00.022S	58°05.010W	22-Feb-06	Susie Lockhart & Vincent Smith	AMLR 2006 - Leg II	USNM 1121295	235	9	EU823176	EU823238	KC246625
G319.4	Bransfield	39-52	62°30.875S	55°58.886W	9-Mar-06	Susie Lockhart & Vincent Smith	AMLR 2006 - Leg II	USNM 1121327	238	10	EU823153	EU823239	KC246626
G313.1	Bransfield	24-14	62°47.906S	57°20.485W	23-Feb-06	Susie Lockhart & Vincent Smith	AMLR 2006 - Leg II	USNM 1121351	164	10	EU823153	EU823244	KC246627
G323.4	Bransfield	31-53	63°03.845S	57°09.281W	10-Mar-06	Susie Lockhart & Vincent Smith	AMLR 2006 - Leg II	USNM 1120835	253	10	EU823153	EU823250	KC246628
G320.1	Bransfield	20-60	62°52.279S	57°53.022W	15-Mar-06	Susie Lockhart & Vincent Smith	AMLR 2006 - Leg II	USNM 1120712	358	11	EU823154	EU823245	KC246629
G326.2	Bransfield	45-41	62°43.535S	55°11.224W	5-Mar-06	Susie Lockhart & Vincent Smith	AMLR 2006 - Leg II	USNM 1120708	167	13	EU823158	EU823240	KC246631
G311.1	Bransfield	27-33	62°45.902S	56°51.748W	3-Mar-06	Susie Lockhart & Vincent Smith	AMLR 2006 - Leg II	USNM 1121305	178	13	EU823157	EU823240	KC246630
G314.1	Bransfield	38-26	62°22.142S	55°37.153W	27-Feb-06	Susie Lockhart & Vincent Smith	AMLR 2006 - Leg II	USNM 1121299	258	14	EU823160	EU823235	KC246632
ZSM2001 2286-1	Bransfield	158-1	S63°04.70'	W57°31.60'	26-Apr-00	Michael Schrödl	EASIZ 3	ZSM2001 2286-1	95	14	EU823160	EU823235	
G233.3	Bransfield	Station 12	S 63°40.145'	W 61°10.047'	23-May-06	Nerida Wilson	LMG06-05	lost	126	14	EU823195	EU823235	KC246633
G317.2	Bransfield	35-19	62°28.906S	56°17.261W	25-Feb-06	Susie Lockhart & Vincent Smith	AMLR 2006 - Leg II	USNM 1121354	344	15	EU823144	EU823243	KC246640
G325.2	Bransfield	76-35	62°49.005S	56°39.477W	3-Mar-06	Susie Lockhart & Vincent Smith	AMLR 2006 - Leg II	USNM 1120723	108	15	EU823166	EU823243	KC246634
G315.1	Bransfield	21-13	62°58.574S	57°37.114W	23-Feb-06	Susie Lockhart & Vincent Smith	AMLR 2006 - Leg II	USNM 1121319	118	15	EU823161	EU823243	KC246641
G253.5	Bransfield	21-192	S 63°08.838'	W 57°07.441'	25-May-06	Nerida Wilson	LMG06-05	USNM 1121589	146	15	EU823165	EU823247	KC246635
G253.6	Bransfield	21-192	S 63°08.838'	W 57°07.441'	25-May-06	Nerida Wilson	LMG06-05	USNM 1121588	146	15	EU823166	EU823243	KC246636
G253.7	Bransfield	21-192	S 63°08.838'	W 57°07.441'	25-May-06	Nerida Wilson	LMG06-05	USNM 1121620	146	15	EU823166	EU823248	KC246637
G253.8	Bransfield	21-192	S 63°08.838'	W 57°07.441'	25-May-06	Nerida Wilson	LMG06-05	USNM 1121594	146	15	EU823166	EU823243	KC246638
G253.9	Bransfield	21-192	S 63°08.838'	W 57°07.441'	25-May-06	Nerida Wilson	LMG06-05	USNM 1121607	146	15	EU823166	EU823243	KC246639
G253.13	Bransfield	21-192	S 63°08.838'	W 57°07.441'	25-May-06	Nerida Wilson	LMG06-05	USNM 1121604	146	16	EU823169	EU823249	KC246642
G324.4	Bransfield	19-11	63°00.022S	58°05.010W	22-Feb-06	Susie Lockhart & Vincent Smith	AMLR 2006 - Leg II	USNM 1120702	235	16	EU823212	EU823253	
G326.4	Bransfield	45-41	62°43.535S	55°11.224W	5-Mar-06	Susie Lockhart & Vincent Smith	AMLR 2006 - Leg II	USNM 1120832	167	17	EU823174	EU823241	KC246643
G322.1	Bransfield	16-9	63°00.513S	58°50.026W	22-Feb-06	Susie Lockhart & Vincent Smith	AMLR 2006 - Leg II	USNM 1120710	353	17	EU823187	EU823241	KC246645
PSC08-06-B	western Peninsula	Jannus	S 64 47.107'	W 64 06.125'	9-Jan-08	Bill Baker & Alan Maschek	SCUBA	destroyed	0-35	17	JX680541	JX683465	KC246644
PSC08-06-D	western Peninsula	Jannus	S 64 47.107'	W 64 06.125'	9-Jan-08	Bill Baker & Alan Maschek	SCUBA	destroyed	0-35	17	JX680542	JX683466	KC246646
ZSM2002 1052	South Georgia	PS61/182-1	S54°27.63'	W35°41.33'	12-Apr-02	Katrin Linse	LAMPOS	ZSM2002 1052	253	18	EU823202	EU823265	KC246647
ZSM2002 802	Elephant Is.	PS61/049-1	S61°11.73'	W54°41.45'	31-Jan-02	Michael Raupach	ANDEEP 1	ZSM2002 802	289	18	EU823140	EU823231	KC246648
G324.2	Bransfield	19-11	63°00.022S	58°05.010W	22-Feb-06	Susie Lockhart & Vincent Smith	AMLR 2006 - Leg II	USNM 1120833	235	18	EU823213	EU823252	
G131.4	Bransfield	Station 49	S63°13'45''	W58°45'20''	6-Dec-04	Nerida Wilson	LMG04-14	USNM 1121622	87	19	EU823182	EU823234	KC246650
G131.8	Bransfield	Station 49	S63°13'45''	W58°45'20''	6-Dec-04	Nerida Wilson	LMG04-14	USNM 1121619	87	19	EU823185	EU823234	KC246649
G316.1	nr Elephant Is.	74-32	61°48.831S	54°00.131W	2-Mar-06	Susie Lockhart & Vincent Smith	AMLR 2006 - Leg II	USNM 1121296	291	20	EU823142	EU823232	KC246651

G243.2	Bransfield	Station 13	S 63°24.961'	W 61°50.484'	23-May-06	Nerida Wilson	LMG06-05	USNM 1121315	132	20	EU823192	EU823256	KC246656
G243.3	Bransfield	Station 13	S 63°24.961'	W 61°50.484'	23-May-06	Nerida Wilson	LMG06-05	USNM 1122432	132	20	EU823192	EU823232	KC246663
G243.4	Bransfield	Station 13	S 63°24.961'	W 61°50.484'	23-May-06	Nerida Wilson	LMG06-05	USNM 1121612	132	20	EU823193	EU823257	KC246653
G243.5	Bransfield	Station 13	S 63°24.961'	W 61°50.484'	23-May-06	Nerida Wilson	LMG06-05	USNM 1122055	132	20	EU823194	EU823258	KC246655
G233.2	Bransfield	Station 12	S 63°40.145'	W 61°10.047'	23-May-06	Nerida Wilson	LMG06-05	USNM 1121312	126	20	EU823142	EU823232	KC246652
PSC08-06-C	western Peninsula	Jannus	S 64 47.107'	W 64 06.125'	9-Jan-08	Bill Baker & Alan Maschek	SCUBA	destroyed	0-35	20	JX680543	JX683467	KC246654
PSC08-06-I	western Peninsula	Jannus	S 64 47.107'	W 64 06.125'	9-Jan-08	Bill Baker & Alan Maschek	SCUBA	destroyed	0-35	20	JX680544	JX683468	KC246657
PSC08-06-V	western Peninsula	Hermit	S 64 48.139	W 64 1.438	21-Jan-08	Bill Baker & Alan Maschek	SCUBA	destroyed	0-35	20	JX680545	JX683469	KC246658
PSC08-06-AB	western Peninsula	Hermit	S 64 48.139	W 64 1.438	29-Jan-08	Bill Baker & Alan Maschek	SCUBA	destroyed	0-35	20	JX680546	JX683470	KC246659
PSC08-06-AM	western Peninsula	SE Bona	S 64 46.754'	W 64 2.657'	9-Feb-08	Bill Baker & Craig Aumack	SCUBA	destroyed	0-35	20	JX680547	JX683471	KC246660
PSC08-06-AO	western Peninsula	Litchfield	S 64 45.972'	W 64 05.964'	10-Feb-08	Bill Baker & Alan Maschek	SCUBA	destroyed	0-35	20	JX680548	JX683472	KC246661
PSC08-06-BC	western Peninsula	Norsel	S 64 45.638'	W 64 05.874'	27-Feb-08	Bill Baker & Alan Maschek	SCUBA	destroyed	0-35	20	JX680549	JX683473	KC246662
ZSM2001 2244	east Weddell Sea	109-1	S71°11.30'	W12°18.50'	4-Apr-00	Michael Schrödl	EASIZ 3	ZSM2001 2244	314	21	EU823198	EU823262	KC246665
ZSM2001 2173	east Weddell Sea	85-1	S71°11.30'	W12°15.40'	2-Apr-00	Michael Schrödl	EASIZ 3	ZSM2001 2173	314	21	EU823201	EU823262	KC246664
ZSM2001 2245	east Weddell Sea	111-16	S71°07.45'	W11°27.64'	5-Apr-00	Martin Rauschert	EASIZ 3	ZSM2001 2245	80	22	EU823199	EU823263	KC246666
ZSM2001 2246	east Weddell Sea	111-16	S71°07.45'	W11°27.64'	5-Apr-00	Martin Rauschert	EASIZ 3	ZSM2001 2246	80	23	EU823200	EU823264	KC246667
G168.101	Shag Rocks	29-OT27	S53°45'00''	W41°28'12''	5-Jun-04	Nerida Wilson	ICEFISH	USNM 1120826	191	24	EU823134	EU823224	KC246673
G168.102	Shag Rocks	29-OT27	S53°45'00''	W41°28'12''	5-Jun-04	Nerida Wilson	ICEFISH	USNM 1120721	191	24	EU823134	EU823225	KC246674
CAS IZ171176	Bouvet Is.	81-OT58	S54°29'24''	W03°17'60''	30-Jun-04	Susie Lockhart	ICEFISH	CAS IZ171176	169	24	EU823203	EU823229	KC246684
ZSM2002 1056	Elephant Is.	PS61/081-1	S61°02.91'	W55°52.78'	7-Feb-02	Michael Schrödl	ANDEEP 1	ZSM2002 1056	149	24	EU823138	EU823229	KC246668
G319.1	Bransfield	39-52	62°30.875S	55°58.886W	9-Mar-06	Susie Lockhart & Vincent Smith	AMLR 2006 - Leg II	USNM 1121357	238	24	EU823150	EU823236	KC246682
G312.1	Bransfield	33-18	62°36.778S	56°36.615W	25-Feb-06	Susie Lockhart & Vincent Smith	AMLR 2006 - Leg II	USNM 1121345	231	24	EU823156	EU823236	KC246688
G323.1	Bransfield	31-53	63°03.845S	57°09.281W	10-Mar-06	Susie Lockhart & Vincent Smith	AMLR 2006 - Leg II	USNM 1120836	253	24	EU823138	EU823236	KC246669
G131.3.01	Bransfield	Station 49	S63°13'45''	W58°45'20''	6-Dec-04	Nerida Wilson	LMG04-14	USNM 1121609	87	24	EU823180	EU823236	KC246681
G131.3.03	Bransfield	Station 49	S63°13'45''	W58°45'20''	6-Dec-04	Nerida Wilson	LMG04-14	USNM 1121613	87	24	EU823138	EU823236	KC246671
G131.3.04	Bransfield	Station 49	S63°13'45''	W58°45'20''	6-Dec-04	Nerida Wilson	LMG04-14	USNM 1121598	87	24	EU823138	EU823236	KC246670
G131.3.05	Bransfield	Station 49	S63°13'45''	W58°45'20''	6-Dec-04	Nerida Wilson	LMG04-14	USNM 1121590	87	24	EU823156	EU823236	KC246689
G131.3.06	Bransfield	Station 49	S63°13'45''	W58°45'20''	6-Dec-04	Nerida Wilson	LMG04-14	USNM 1121597	87	24	EU823181	EU823236	KC246683
G131.5	Bransfield	Station 49	S63°13'45''	W58°45'20''	6-Dec-04	Nerida Wilson	LMG04-14	USNM 1121621	87	24	EU823183	EU823236	KC246675
G131.6	Bransfield	Station 49	S63°13'45''	W58°45'20''	6-Dec-04	Nerida Wilson	LMG04-14	USNM 1121591	87	24	EU823129	EU823236	KC246672
G131.7	Bransfield	Station 49	S63°13'45''	W58°45'20''	6-Dec-04	Nerida Wilson	LMG04-14	USNM 1121602	87	24	EU823184	EU823236	KC246678
G266.1	Bransfield	Station 22	S 63°07.150'	W 58°41.522'	26-May-06	Nerida Wilson	LMG06-05	USNM 1121605	150	24	EU823156	EU823236	KC246685
G266.2	Bransfield	Station 22	S 63°07.150'	W 58°41.522'	26-May-06	Nerida Wilson	LMG06-05	USNM 1121601	150	24	EU823156	EU823236	KC246686
G137.4.01	Bransfield	Station 51	S63°23'03''	W60°03'24''	6-Dec-04	Nerida Wilson	LMG04-14	USNM 1120718	277	24	EU823156	EU823236	KC246687
G149.1	Bransfield	Station 67	S63°09'60''	W62°24'29''	9-Dec-04	Nerida Wilson	LMG04-14	USNM 1121611	192	24	EU823196	EU823259	KC246679
G149.2	Bransfield	Station 67	S63°09'60''	W62°24'29''	9-Dec-04	Nerida Wilson	LMG04-14	lost	192	24	EU823197	EU823260	KC246680
PSC08-06-A	western Peninsula	Jannus	S 64 47.107'	W 64 06.125'	8-Jan-08	Bill Baker & Alan Maschek	SCUBA	destroyed	0-35	24	JX680552	JX683476	KC246676
PSC08-06-J	western Peninsula	Jannus	S 64 47.107'	W 64 06.125'	11-Jan-08	Bill Baker & Craig Aumack	SCUBA	destroyed	0-35	24	JX680553	JX683477	KC246677
PSC08-06-U	western Peninsula	Hermit	S 64 48.139	W 64 1.438	21-Jan-08	Bill Baker & Alan Maschek	SCUBA	destroyed	0-35	24	JX680550	JX683474	KC246690
PSC08-06-Z	western Peninsula	Hermit	S 64 48.139	W 64 1.438	29-Jan-08	Bill Baker & Alan Maschek	SCUBA	destroyed	0-35	24	JX680554	JX683478	KC246692
PSC08-06-AA	western Peninsula	Hermit	S 64 48.139	W 64 1.438	29-Jan-08	Bill Baker & Alan Maschek	SCUBA	destroyed	0-35	24	JX680551	JX683475	KC246691
G173	Ross Sea	New Harbour	S77°34.302'	E163°30.702'	Nov 2005	Sam Bowser	SCUBA	USNM 1121603	0-35	25	EU823207	EU823267	KC246702
G174	Ross Sea	New Harbour	S77°34.302'	E163°30.702'	Nov 2005	Sam Bowser	SCUBA	USNM 1121583	0-35	25	EU823207	EU823267	KC246701
G175	Ross Sea	New Harbour	S77°34.302'	E163°30.702'	Nov 2005	Sam Bowser	SCUBA	USNM 1121584	0-35	25	EU823207	EU823267	KC246700
G176	Ross Sea	New Harbour	S77°34.302'	E163°30.702'	Nov 2005	Sam Bowser	SCUBA	USNM 1121600	0-35	25	EU823207	EU823267	KC246704
G177	Ross Sea	New Harbour	S77°34.302'	E163°30.702'	Nov 2005	Sam Bowser	SCUBA	USNM 1121615	0-35	25	EU823208	EU823267	KC246693
G178	Ross Sea	New Harbour	S77°34.302'	E163°30.702'	Nov 2005	Sam Bowser	SCUBA	USNM 1121585	0-35	25	EU823208	EU823268	KC246703
G179	Ross Sea	New Harbour	S77°34.302'	E163°30.702'	Nov 2005	Sam Bowser	SCUBA	USNM 1121586	0-35	25	EU823207	EU823267	KC246699
G180	Ross Sea	New Harbour	S77°34.302'	E163°30.702'	Nov 2005	Sam Bowser	SCUBA	USNM 1121614	0-35	25	EU823207	EU823267	KC246698
G182	Ross Sea	New Harbour	S77°34.302'	E163°30.702'	Nov 2005	Sam Bowser	SCUBA	USNM 1121623	0-35	25	EU823208	EU823267	KC246697
G183	Ross Sea	New Harbour	S77°34.302'	E163°30.702'	Nov 2005	Sam Bowser	SCUBA	USNM 1121580	0-35	25	EU823207	EU823267	KC246696
G184	Ross Sea	New Harbour	S77°34.302'	E163°30.702'	Nov 2005	Sam Bowser	SCUBA	USNM 1121582	0-35	25	EU823207	EU823267	KC246695
G185	Ross Sea	New Harbour	S77°34.302'	E163°30.702'	Nov 2005	Sam Bowser	SCUBA	USNM 1121587	0-35	25	EU823208	EU823267	KC246694
G280.1	western Peninsula	Station 33	S 67°44.420'	W 69°17.379'	30-May-06	Nerida Wilson	LMG06-05	USNM 1122433	122	27	EU823210	EU823261	KC246705
PSC08-06-H	western Peninsula	Jannus	S 64 47.107'	W 64 06.125'	9-Jan-08	Bill Baker & Alan Maschek	SCUBA	destroyed	0-35	27	JX680555	JX683479	KC246706
G214.1	Burdwood Bank	Station 6	S 54°49'	W 60°16'	16-May-06	Nerida Wilson	LMG06-05	USNM 1121608	110	28	EU823127	EU823219	KC246709
G85.1	Burdwood Bank	Station 14	S54°41'25''	W59°23'31''	16-May-06	Nerida Wilson	LMG04-14	USNM 1120707	207	28	EU823128	EU823220	KC246708
CAS IZ171180b	Burdwood Bank	9-BT7	S54°31'12''	W56°37'12''	22-May-04	Susie Lockhart	ICEFISH	CAS IZ171180b	125	28	EU823129	EU823220	KC246707
ZSM2002 1249	Burdwood Bank	PS61/150-1	S54°30.22'	W56°08.20'	6-Apr-02	Michael Schrödl	LAMPOS	ZSM2002 1249	289	28	EU823217	EU823222	
ZSM2002 1059-1	Burdwood Bank	PS61/153-1	S54°31.22'	W56°08.93'	6-Apr-02	Katrin Linse	LAMPOS	ZSM2002 1059-1	287	28	EU823205	EU823220	
ZSM2002 1059-3	Burdwood Bank	PS61/153-1	S54°31.22'	W56°08.93'	6-Apr-02	Katrin Linse	LAMPOS	ZSM2002 1059-3	287	28	EU823206	EU823266	
G318.1	Bransfield	46-23	62°32.720S	55°21.951W	26-Mar-06	Susie Lockhart & Vincent Smith	AMLR 2006 - Leg II	USNM 1122216	149	29	EU823146	EU823223	KC246742
G318.2	Bransfield	46-23	62°32.720S	55°21.951W	26-Mar-06	Susie Lockhart & Vincent Smith	AMLR 2006 - Leg II	USNM 1121322	149	29	EU823214	EU823223	KC246711
G318.3	Bransfield	46-23	62°32.720S	55°21.951W	26-Mar-06	Susie Lockhart & Vincent Smith	AMLR 2006 - Leg II	USNM 1121331	149	29	EU823147	EU823223	
G318.5	Bransfield	46-23	62°32.720S	55°21.951W	26-Mar-06	Susie Lockhart & Vincent Smith	AMLR 2006 - Leg II	USNM 1121313	149	29	EU823149	EU823221	KC246741
G326.3	Bransfield	45-41	62°43.535S	55°11.224W	5-Mar-06	Susie Lockhart & Vincent Smith	AMLR 2006 - Leg II	USNM 1120720	167	29	EU823159	EU823223	KC246730
G325.7	Bransfield	76-35	62°49.005S	56°39.477W	3-Mar-06	Susie Lockhart & Vincent Smith	AMLR 2006 - Leg II	USNM 1120719	108	29	EU823146	EU823223	KC246712
G253.1	Bransfield	21-192	S 63°08.838'	W 57°07.441'	25-May-06	Nerida Wilson	LMG06-05	USNM 1121592	146	29	EU823162	EU823223	
G253.2	Bransfield	21-192	S 63°08.838'	W 57°07.441'	25-May-06	Nerida Wilson	LMG06-05	USNM 1121593	146	29	EU823163	EU823223	KC246710

G253.3	Bransfield	21-192	S 63˚08.838'	W 57˚07.441'	25-May-06	Nerida Wilson	LMG06-05	USNM 1121606	146	29	EU823164	EU823223	
G253.4	Bransfield	21-192	S 63˚08.838'	W 57˚07.441'	25-May-06	Nerida Wilson	LMG06-05	USNM 1121610	146	29	EU823163	EU823246	KC246734
G253.10	Bransfield	21-192	S 63˚08.838'	W 57˚07.441'	25-May-06	Nerida Wilson	LMG06-05	USNM 1121596	146	29	EU823162	EU823223	KC246731
G253.12	Bransfield	21-192	S 63˚08.838'	W 57˚07.441'	25-May-06	Nerida Wilson	LMG06-05	USNM 1121616	146	29	EU823168	EU823223	KC246733
G253.14.02	Bransfield	21-192	S 63˚08.838'	W 57˚07.441'	25-May-06	Nerida Wilson	LMG06-05	USNM 1121581	146	29	EU823170	EU823223	KC246732
ZSM2001 2286-7	Bransfield	158-1	S63°04.70'	W57°31.60'	26-Apr-00	Michael Schrödl	EASIZ 3	ZSM2001 2286-7	95	29	EU823218	EU823223	
ZSM2001 2307-1	Bransfield	158-1	S63°04.70'	W57°31.60'	26-Apr-00	Michael Schrödl	EASIZ 3	ZSM2001 2307-1	95	29	EU823159	EU823223	KC246753
ZSM2001 2307-2	Bransfield	158-1	S63°04.70'	W57°31.60'	26-Apr-00	Michael Schrödl	EASIZ 3	ZSM2001 2307-2	95	29	EU823171	EU823223	KC246752
ZSM2001 2307-3	Bransfield	158-1	S63°04.70'	W57°31.60'	26-Apr-00	Michael Schrödl	EASIZ 3	ZSM2001 2307-3	95	29	EU823172	EU823223	KC246751
ZSM2001 2307-4	Bransfield	158-1	S63°04.70'	W57°31.60'	26-Apr-00	Michael Schrödl	EASIZ 3	ZSM2001 2307-4	95	29	EU823173	EU823251	KC246750
G324.7	Bransfield	19-11	63˚00.022S	58˚05.010W	22-Feb-06	Susie Lockhart & Vincent Smith	AMLR 2006 - Leg II	USNM 1121348	235	29	EU823215	EU823223	
G137.2	Bransfield	Station 51	S63°23'03''	W60°03'24''	6-Dec-04	Nerida Wilson	LMG04-14	USNM 1120716	277	29	EU823163	EU823223	KC246749
G137.4.02	Bransfield	Station 51	S63°23'03''	W60°03'24''	6-Dec-04	Nerida Wilson	LMG04-14	USNM 1120705	277	29	EU823188	EU823246	KC246735
G137.4.03	Bransfield	Station 51	S63°23'03''	W60°03'24''	6-Dec-04	Nerida Wilson	LMG04-14	USNM 1120713	277	29	EU823189	EU823223	KC246736
G137.4.04	Bransfield	Station 51	S63°23'03''	W60°03'24''	6-Dec-04	Nerida Wilson	LMG04-14	USNM 1120824	277	29	EU823190	EU823246	KC246745
G137.5.02	Bransfield	Station 51	S63°23'03''	W60°03'24''	6-Dec-04	Nerida Wilson	LMG04-14	USNM 1121291	277	29	EU823163	EU823223	KC246746
G137.5.03	Bransfield	Station 51	S63°23'03''	W60°03'24''	6-Dec-04	Nerida Wilson	LMG04-14	USNM 1120704	277	29	EU823163	EU823223	KC246744
G137.5.04	Bransfield	Station 51	S63°23'03''	W60°03'24''	6-Dec-04	Nerida Wilson	LMG04-14	lost	277	29	EU823163	EU823246	KC246743
G137.6	Bransfield	Station 51	S63°23'03''	W60°03'24''	6-Dec-04	Nerida Wilson	LMG04-14	lost	277	29	EU823191	EU823223	KC246747
G137.7	Bransfield	Station 51	S63°23'03''	W60°03'24''	6-Dec-04	Nerida Wilson	LMG04-14	lost	277	29	EU823163	EU823246	KC246737
G137.8	Bransfield	Station 51	S63°23'03''	W60°03'24''	6-Dec-04	Nerida Wilson	LMG04-14	lost	277	29	EU823163	EU823223	KC246738
G137.9	Bransfield	Station 51	S63°23'03''	W60°03'24''	6-Dec-04	Nerida Wilson	LMG04-14	lost	277	29	EU823163	EU823223	KC246739
G137.10	Bransfield	Station 51	S63°23'03''	W60°03'24''	6-Dec-04	Nerida Wilson	LMG04-14	lost	277	29	EU823163	EU823255	KC246740
PSC08-06-E	western Peninsula	Jannus	S 64 47.107'	W 64 06.125'	9-Jan-08	Bill Baker & Alan Maschek	SCUBA	destroyed	0-35	29	JX680573	JX683497	KC246716
PSC08-06-F	western Peninsula	Jannus	S 64 47.107'	W 64 06.125'	9-Jan-08	Bill Baker & Alan Maschek	SCUBA	destroyed	0-35	29	JX680574	JX683498	KC246715
PSC08-06-G	western Peninsula	Jannus	S 64 47.107'	W 64 06.125'	9-Jan-08	Bill Baker & Alan Maschek	SCUBA	destroyed	0-35	29	JX680575	JX683499	KC246714
PSC08-06-K	western Peninsula	Bonaparte	S 64 46.662'	W 64 03.986'	12-Jan-08	Bill Baker & Alan Maschek	SCUBA	destroyed	0-35	29	JX680576	JX683500	KC246759
PSC08-06-L	western Peninsula	Bonaparte	S 64 46.662'	W 64 03.986'	12-Jan-08	Bill Baker & Alan Maschek	SCUBA	destroyed	0-35	29	JX680577	JX683501	KC246758
PSC08-06-M	western Peninsula	Bonaparte	S 64 46.662'	W 64 03.986'	12-Jan-08	Bill Baker & Alan Maschek	SCUBA	destroyed	0-35	29	JX680578	JX683502	KC246760
PSC08-06-Q	western Peninsula	**Bonaparte**	S 64 46.662'	W 64 03.986'	13-Jan-08	Bill Baker, Craig Aumack & Alan Maschek	SCUBA	destroyed	0-35	29	JX680579	JX683503	KC246757
PSC08-06-AC	western Peninsula	Hermit	S 64 48.139	W 64 1.438	29-Jan-08	Bill Baker & Alan Maschek	SCUBA	destroyed	0-35	29	JX680556	JX683480	KC246755
PSC08-06-AF	western Peninsula	Hermit	S 64 48.139	W 64 1.438	29-Jan-08	Bill Baker & Alan Maschek	SCUBA	destroyed	0-35	29	JX680557	JX683481	KC246717
PSC08-06-AK	western Peninsula	Laggard	S 64 48.574'	W 64 0.986'	5-Feb-08	Bill Baker & Craig Aumack	SCUBA	destroyed	0-35	29	JX680558	JX683482	KC246729
PSC08-06-AN	western Peninsula	SE Bona	S 64 46.754'	W 64 2.657'	9-Feb-08	Bill Baker & Craig Aumack	SCUBA	destroyed	0-35	29	JX680559	JX683483	KC246728
PSC08-06-AT	western Peninsula	Hero Inlet	S 64 46.473'	W 64 03.284'	13-Feb-08	Bill Baker & Alan Maschek	SCUBA	destroyed	0-35	29	JX680560	JX683484	KC246727
PSC08-06-AU	western Peninsula	Hero Inlet	S 64 46.473'	W 64 03.284'	13-Feb-08	Bill Baker & Alan Maschek	SCUBA	destroyed	0-35	29	JX680561	JX683485	KC246726
PSC08-06-AV	western Peninsula	Hero Inlet	S 64 46.473'	W 64 03.284'	13-Feb-08	Bill Baker & Alan Maschek	SCUBA	destroyed	0-35	29	JX680562	JX683486	KC246725
PSC08-06-AW	western Peninsula	Hero Inlet	S 64 46.473'	W 64 03.284'	13-Feb-08	Bill Baker & Alan Maschek	SCUBA	destroyed	0-35	29	JX680563	JX683487	KC246724
PSC08-06-AZ	western Peninsula	Hero Inlet	S 64 46.473'	W 64 03.284'	13-Feb-08	Bill Baker & Alan Maschek	SCUBA	destroyed	0-35	29	JX680564	JX683488	KC246723
PSC08-06-BA	western Peninsula	Hero Inlet	S 64 46.473'	W 64 03.284'	13-Feb-08	Bill Baker & Alan Maschek	SCUBA	destroyed	0-35	29	JX680565	JX683489	KC246754
PSC08-06-BK	western Peninsula	Hero Inlet	S 64 46.473'	W 64 03.284'	3-Mar-08	Bill Baker & Charles Amsler	SCUBA	destroyed	0-35	29	JX680572	JX683496	KC246722
PSC08-06-BD	western Peninsula	Norsel	S 64 45.638'	W 64 05.874'	27-Feb-08	Bill Baker & Alan Maschek	SCUBA	destroyed	0-35	29	JX680566	JX683490	KC246721
PSC08-06-BE	western Peninsula	Norsel	S 64 45.638'	W 64 05.874'	27-Feb-08	Bill Baker & Alan Maschek	SCUBA	destroyed	0-35	29	JX680567	JX683491	KC246720
PSC08-06-BF	western Peninsula	Norsel	S 64 45.638'	W 64 05.874'	27-Feb-08	Bill Baker & Alan Maschek	SCUBA	destroyed	0-35	29	JX680568	JX683492	KC246756
PSC08-06-BG	western Peninsula	Step stone	S 64 47.190'	W 63 59.830	2-Mar-08	Bill Baker & Alan Maschek	SCUBA	destroyed	0-35	29	JX680569	JX683493	KC246719
PSC08-06-BH	western Peninsula	Step stone	S 64 47.190'	W 63 59.830	2-Mar-08	Bill Baker & Alan Maschek	SCUBA	destroyed	0-35	29	JX680570	JX683494	KC246718
PSC08-06-BJ	western Peninsula	Step stone	S 64 47.190'	W 63 59.830	2-Mar-08	Bill Baker & Alan Maschek	SCUBA	destroyed	0-35	29	JX680571	JX683495	KC246713
PSC08-06-AD	western Peninsula	Hermit	S 64 48.139	W 64 1.438	29-Jan-08	Bill Baker & Alan Maschek	SCUBA	destroyed	0-35	30	JX680580	JX683504	KC246764
PSC08-06-BI	western Peninsula	Step stone	S 64 47.190'	W 63 59.830	2-Mar-08	Bill Baker & Alan Maschek	SCUBA	destroyed	0-35	30	JX680583	JX683507	KC246761
PSC08-06-AI	western Peninsula	step stone	S 64 47.190'	W 63 59.830	4-Feb-08	Bill Baker & Alan Maschek	SCUBA	destroyed	0-35	30	JX680581	JX683505	KC246763
PSC08-06-AJ	western Peninsula	step stone	S 64 47.190'	W 63 59.830	4-Feb-08	Bill Baker & Alan Maschek	SCUBA	destroyed	0-35	30	JX680582	JX683506	KC246762
PSC08-06-T	western Peninsula	Gamage	S 64 46.476'	W 64 03.415'	18-Jan-08	Alan Maschek & Craig Aumack	SCUBA	destroyed	0-35	31	JX680584	JX683508	KC246765
PSC08-06-W	western Peninsula	Hermit	S 64 48.139	W 64 1.438	21-Jan-08	Bill Baker & Alan Maschek	SCUBA	destroyed	0-35	31	JX680585	JX683509	KC246766
PSC08-06-R	western Peninsula	Gamage	S 64 46.476'	W 64 03.415'	18-Jan-08	Alan Maschek & Craig Aumack	SCUBA	destroyed	0-35	32	JX680586	JX683510	KC246769
PSC08-06-AS	western Peninsula	Hero Inlet	S 64 46.473'	W 64 03.284'	13-Feb-08	Bill Baker & Alan Maschek	SCUBA	destroyed	0-35	32	JX680587		KC246767
PSC08-06-AX	western Peninsula	Hero Inlet	S 64 46.473'	W 64 03.284'	13-Feb-08	Bill Baker & Alan Maschek	SCUBA	destroyed	0-35	32	JX680588	JX683511	KC246768
PSC08-06-AY	western Peninsula	Hero Inlet	S 64 46.473'	W 64 03.284'	13-Feb-08	Bill Baker & Alan Maschek	SCUBA	destroyed	0-35	32	JX680589	JX683512	KC246770

Localities in bold have a query on their finescale preciseness, +/- 1km
