# Supplementary material for: A Species Flock Driven by Predation? Secondary Metabolites Support Diversification of Slugs in Antarctica
Source: PLoS One. 2013 Nov 26;8(11):e80277. doi: 10.1371/journal.pone.0080277 (PMC3841181; doi:10.1371/journal.pone.0080277)
Supplement: Table S2 — Geographical and depth distribution of diterpenes found in Doris kerguelenensis . Synonymous compounds are reported in rounded parentheses, and geographical detail in square parentheses. (PDF) [file pone.0080277.s005.pdf]

Supplementary Table 2. Geographical and depth distribution of diterpenes found in *Doris kerguelenensis.* Synonymous compounds are reported in rounded parentheses, and geographical detail in square parentheses.

Current accepted structure	Reference	Depth	halimane	labdane	isocopalane	clerodane
Compound 1	Gavagnin et al. Tet Assym 1999	-	West Antarctic Peninsula [South Livingston Is.]			
Compound 8	Gavagnin et al. Tet 2003	10-20m	Ross Sea [Terra Nova Bay]			
(reported as compound 3)	Gavagnin et al. Tet Assym 1999	70m	West Antarctic Peninsula [South Livingston Is.]			
(reported as compound 3a)	Cutigano et al. Eur J Org Chem 2011	3-15m; 286-848m	Ross Sea [Terra Nova Bay]; Weddell Sea			
Austrodorin compound 1	Gavagnin et al. Tett Lett 1995	70m	Ross Sea [Tethys Bay]			
Compound 12	Gavagnin et al. Tet 2003	10-20m	Ross Sea [Terra Nova Bay]			
(reported as compound 3b)	Cutigano et al. Eur J Org Chem 2011	3-15m; 286-848m	Ross Sea [Terra Nova Bay]; Weddell Sea			
Palmadorin R	Maschek et al.	1-40m	West Antarctic Peninsula [Anvers Is]			

Palmadorin S	Maschek et al.	1-40m	West Antarctic Peninsula<br>[Anvers Is]	
Compound 7	Gavagnin et al. Tet 2003	10-20m		Ross Sea<br>[Terra Nova Bay]
(reported as compound 1)	Davies-Coleman & Faulkner<br>Tet 1991	25-35 m		Ross Sea<br>[McMurdo Sound]
(reported as compound 4)	Gavagnin et al.<br>Tet Assym 1999	10-20m		Weddell Sea
(reported as compound 1a)	Cutigano et al. Eur J Org Chem 2011	3-15m;<br>286-848m		Ross Sea<br>[Terra Nova Bay];<br>Weddell Sea
Compound 4	Davies-Coleman & Faulkner<br>Tet 1991	25-35m		Ross Sea<br>[McMurdo Sound]
(reported as compound 2)	Gavagnin et al.<br>Tet Assym 1999	trawl		Weddell Sea
Compound 8	Davies-Coleman & Faulkner<br>Tet 1991	25-35m		Ross Sea<br>[McMurdo Sound]
Compound 6a	Cutigano et al. 2011	3-15m		Ross Sea<br>[Terra Nova Bay]
Compound 6b	Cutigano et al. 2011	3-15m		Ross Sea<br>[Terra Nova Bay]
Compound 7a	Cutigano et al. 2011	3-15m		Ross Sea<br>[Terra Nova Bay]

(reported as Palmadorin O	Maschek et al.	3-15m	West Antarctic Peninsula [Anvers Is]		
Compound 7b	Cutigano et al.2011	3-15m	Ross Sea [Terra Nova Bay]		
(reported as Palmadorin M)	Maschek et al.	2-40m	West Antarctic Peninsula [Anvers Is]		
Palmadorin N	Maschek et al.	1-40m	West Antarctic Peninsula [Anvers Is]		
Palmadorin P	Maschek et al.	1-40m	West Antarctic Peninsula [Anvers Is]		
Palmadorin Q	Maschek et al.	1-40m	West Antarctic Peninsula [Anvers Is]		
Austrodorin-A, compound 7	Gavagnin et al. Tett Lett 1999	-		West Antarctic Peninsula [South Shetland Is]	
Austrodorin-B, compound 8	Gavagnin et al. Tett Lett 1999	-		West Antarctic Peninsula [South Shetland Is]	
Compound 4	Cutigano et al.2011	3-15m		Ross Sea [Terra Nova Bay]	
Compound 9	Gavagnin et al. Tet 2003	10-20m			Ross Sea [Terra Nova Bay]
Palmodorin A, Compound 1	Diyabalange et al. JNP 2011	1-40m			West Antarctic Peninsula [Anvers Is]

Palmodorin B, Compound 2	Diyabalange et al. JNP 2011	1-40m	West Antarctic Peninsula [Anvers Is]
Palmodorin C, Compound 3	Diyabalange et al. JNP 2011	1-40m	West Antarctic Peninsula [Anvers Is]
Compound 5	Cutigano et al.2011	3-15m	Ross Sea [Terra Nova Bay]
Palmadorin D	Maschek et al.	1-40m	West Antarctic Peninsula [Anvers Is]
Palmadorin E	Maschek et al.	1-40m	West Antarctic Peninsula [Anvers Is]
Palmadorin F	Maschek et al.	1-40m	West Antarctic Peninsula [Anvers Is]
Palmadorin G	Maschek et al.	1-40m	West Antarctic Peninsula [Anvers Is]
Palmadorin H	Maschek et al.	1-40m	West Antarctic Peninsula [Anvers Is]
Palmadorin I	Maschek et al.	1-40m	West Antarctic Peninsula [Anvers Is]
Palmadorin J	Maschek et al.	1-40m	West Antarctic Peninsula [Anvers Is]
Palmadorin K	Maschek et al.	1-40m	West Antarctic Peninsula

			[Anvers Is]
Palmadorin L	Maschek et al.	1-40m	West Antarctic Peninsula<br>[Anvers Is]

Note: Compounds 1b and 2 from Cutigano et al. 2012 cannot be verified from sources given.
